# Facebook Support Groups for Pediatric Rare Diseases: Cross-Sectional Study to Investigate Opportunities, Limitations, and Privacy Concerns

**DOI:** 10.2196/31411

**Published:** 2022-01-06

**Authors:** Sarah Catrin Titgemeyer, Christian P Schaaf

**Affiliations:** 1 University of Cologne Cologne Germany; 2 Institute of Human Genetics Heidelberg University Heidelberg Germany

**Keywords:** Facebook, support group, parental support, pediatric rare diseases, privacy paradox, children’s privacy

## Abstract

**Background:**

Because of the nature of rare diseases with affected individuals being widely geographically dispersed, finding an in-person/offline support group itself can be a challenge. Affected individuals therefore turn to social networking platforms such as Facebook for online support groups.

**Objective:**

We aim to put into perspective the opportunities Facebook offers as a tool for pediatric rare disease support groups by investigating its use, advantages, and limitations including privacy concerns. We analyze group accessibility and usage, advantages specific to rare diseases, perceived privacy, and views on using Facebook for communication between health professionals and parents, pharmaceutical companies, and study recruitment.

**Methods:**

We contacted 12 Facebook support groups for 12 respective rare diseases with pediatric onset and invited group members to participate in a cross-sectional online survey.

**Results:**

Of 231 respondents, 87.0% (n=201) of respondents were female, 12.6% (n=29) were male, and 0.4% reported another sex (n=1). Respondents’ mean age was 41.56 years (SD 9.375); 91.3% (n=211) of respondents were parents (183 mothers, 27 fathers, 1 other sex); 59.7% (n=138) reported a self-initiated search for the Facebook group, 24.2% (n=56) received recommendations from their health professionals, and 12.6% (n=29) recommendations from someone else affected by the disease. On average, support group members visited Facebook at least once a day, visited and passively participated (read/liked posts) several times a week, and participated actively (commented/posted) once a month. As much as 79.2% (183/231) agreed that they would like to have health professionals as members of the respective Facebook group. Group members expressed more concern about privacy issues on Facebook in general than in their respective Facebook support groups, with concerns mostly related to Facebook itself and nongroup members.

**Conclusions:**

Our study confirmed that Facebook enhances support group accessibility for parents of children with rare diseases. Group participants perceive a reduction and elimination of distance, a common challenge in rare disease, and Facebook support groups create an environment of perceived privacy. The group’s privacy setting can be a critical factor for active support group participation. Sharing personal information and pictures on Facebook is very common among group participants, which shows the importance of discussing and protecting children’s privacy rights in this context.

**Trial Registration:**

German Clinical Trials Register DRKS00016067; https://www.drks.de/drks_web/navigate.do?navigationId=trial.HTML&TRIAL_ID=DRKS00016067

## Introduction

The types of emotions most frequently expressed by parents of children with a rare disease include fear, worry, frustration, uncertainty, helplessness, and vulnerability [1]. Parents often feel dissatisfied with the overall level of support for their child with a rare disease [1]. Affected individuals are often geographically dispersed, because rare diseases by definition have a very low prevalence. According to the European definition, a disease is considered rare when its prevalence is below 1 in 2000 [2]. Therefore, many parents have never come into contact with another parent taking care of a child with a similar condition [1]. Social isolation and the feeling of being disconnected from society are common experiences [3]. For many rare diseases, medical and scientific knowledge remains scarce [2]. At the same time, these diseases are often serious and chronic, thereby increasing psychological, social, economic, and cultural vulnerability [2]. Consequently, parents of children with rare diseases encounter substantial challenges and have special supportive care needs [1,3].

Support groups offer improved social support [1,4,5] through befriending other people with similar experiences; learning about the disease, treatments, and coping skills; emotional support; and feeling empowered [4]. However, due to the nature of rare diseases with affected individuals being widely geographically dispersed, finding an in-person/offline support group itself can be a challenge. Affected individuals therefore turn to social networking platforms such as Facebook for online support groups. The use of social media for health communication offers increased interactions; more available, shared, and tailored information; and peer, emotional, and social support [6].

Facebook is one of the longest existing social networking platforms [7], and with more than 2.4 billion monthly active users also one of the largest [8]. In March 2019, Facebook support groups were available for more than 4000 pediatric rare diseases with approximately 1.8 million group members in more than 6000 groups [9]. Facebook has an international scope, provides options for individual and group communication [7], offers an unlimited number of participants, and is very cost-effective [10]. Nevertheless, Facebook support group accessibility is limited by access to a computer and the internet [10], related handling skills, and age restrictions. Concerning informational exchanges on social media, the reliability [6], accuracy [10], quality [11], application to personal situations [6], and the possible misinterpretation [10] of information found online and on social media have been questioned. Especially considering how frequently Facebook is being used for support groups, an investigation into whether Facebook represents a suitable tool for pediatric rare disease support groups is needed to improve the much needed support for parents of children with rare diseases.

The usage of Facebook for parent support groups can involve sharing a child’s personal health information online. Sharing information online can be potentially harmful due to the ability to identify individuals and the potential misuse of this information by organizations and individuals [12]. Known negative consequences of sharing information about a child on social media include embarrassment, humiliation, and bullying [13]. The evolvement of sharing private information about children online thus encloses a controversial discussion on a child’s digital identity and protecting children’s rights online [13].

Integrative privacy theories define privacy as a right that should be protected and as individual control of personal information in the form of restricted access [14,15]. Information and communication have been identified as the most relevant dimension of privacy when discussing internet privacy [16]. While the United Nations’ Convention on the Rights of the Child protects children’s privacy, honor, and reputation [17], only little guidance is provided by specific privacy laws regarding children’s need for protection from their parents’ online disclosure [13]. When considering how to protect a child’s privacy online, different approaches to decision making on online information disclosure can play a role, such as decision making based on risk–benefit calculations or decision making based on benefits with little to no risk assessment [18]. A discrepancy between expressed privacy concerns and actual information disclosure, which often becomes evident in online communication, is described as the privacy paradox [18].

It is important to analyze how privacy dimensions, approaches to decision making on online information disclosure, and the privacy paradox play a role in the use of Facebook as a tool for pediatric rare disease support groups to improve the protection of children’s privacy rights and awareness of the risks related to sharing information online.

The role of health professionals in Facebook support groups has not yet been defined. To date, only few studies have examined the opportunities Facebook offers for a communication between parents and health professionals, pharmaceutical companies, and study recruitment [19-24]. Social media can improve patient-to-patient and patient-to-health professional dialogue and can be used for data collection, intervention, promotion, and education [6]. A study on support groups for autism spectrum disorder showed that parents whose diagnosing clinician had referred them to a support group were more likely support group participants [25]. Furthermore, Facebook has been successfully used for study recruitment in rare diseases, resulting in high numbers of study participants with low associated costs, thus improving recruitment for rare disease research [21]. Social media can be used for recruitment of geographically dispersed [20] and socially and culturally diverse [19] individuals. Given all these opportunities, it is of interest to gain insights into group participants’ perspective toward involvement of health professionals and the instrumentalization of Facebook for study recruitment and by pharmaceutical companies.

Only little research exploring the topic of online support groups for pediatric rare diseases has been conducted so far. A few analyses of specific online and Facebook support groups have been performed, for example, on groups for cleft lip and palate [22], clubfoot [5], Hirschsprung disease [23], and autism spectrum disorders [24,25]. These analyses have shown support group benefits which can be classified into the following 3 main categories: informational support, emotional support, and connecting with others. Our prior large quantitative analysis regarding the extent of Facebook support groups for pediatric rare diseases has shown that both the total number of support groups and the number of diseases for which a support group can be found have increased [9]. With two-thirds of these groups being private Facebook groups, we found that the need for privacy should be further explored [9]. Also, given the already widespread use of Facebook as a tool for support groups for pediatric rare diseases, an analysis of its strengths and limitations could allow health professionals to improve their understanding of this tool and, consequently, use Facebook more meaningfully in their counseling and guidance of affected individuals and their family members [9].

With this study, we therefore aim to put into perspective the opportunities Facebook offers as a tool for pediatric rare disease support groups by investigating its use, advantages, and limitations including privacy concerns. We analyze group accessibility and usage, advantages specific to rare diseases, perceived privacy, and views on using Facebook for communication between health professionals and parents, pharmaceutical companies, and study recruitment.

Our results can offer improved knowledge about the opportunities of Facebook support groups as well as their disadvantages. These findings may allow Facebook and similar social media platforms to discover starting points for improving their toolkits and offerings. Parents and caretakers of children with rare diseases can directly or indirectly benefit from this increase in information directly or indirectly through receiving guidance on important points to be considered prior to joining a group from their treating physicians when searching for ways to receive much-needed social support. By informing medical professionals and, subsequently, parents about potential privacy concerns, active decision making on online information disclosure considering children’s privacy rights can be initiated.

## Methods

We contacted 12 Facebook support groups for 12 respective rare diseases with pediatric onset and invited group members to participate in a cross-sectional online survey. For each of these diseases a Facebook group was contacted (Table 1). Group administrators were contacted by either email or Facebook messenger. The members of the respective groups were subsequently invited through a wall post within the actual closed Facebook groups.

The date of first enrolment was July 19, 2019, while the survey was closed on October 10, 2019. Respondents had to be group members of Facebook support groups for rare diseases with childhood onset; this inclusion criterion had to be confirmed in the questionnaire.

**Table 1:** List of each disease for which a Facebook group was contacted.

Orphanet disease description	Orphanet disease synonym	ORPHAcode	OMIM^a^ number
15q13.3 Microdeletion syndrome	Del(15)(q13.3)	199318	612001
Lamb–Shaffer syndrome	SOX5 haploinsufficiency syndrome	530983	616803
Alacrimia–choreoathetosis–liver dysfunction syndrome	NGLY1 deficiency	404454	615273
Optic atrophy–intellectual disability syndrome	BBSOAS	401777	615722
17p11.2 microduplication syndrome	Potocki–Lupski syndrome	1713	610883
Prader–Willi syndrome	Prader–Labhart–Willi syndrome	739	610883
Rare nonsyndromic intellectual disability	CHAMP1 variant	101685	616579
Rett syndrome	—	778	312750
MAGEL2-related Prader–Willi-like syndrome	Schaaf–Yang syndrome	398069	615547
Smith–Magenis syndrome	17p11.2 microdeletion syndrome	819	182290
16p13.2 Microdeletion syndrome	Del(16)(p13.2)	500055	602519
White–Sutton syndrome	Intellectual disability-microcephaly-strabismus-behavioral abnormalities syndrome	468678	616364

^a^OMIM: Online Mendelian Inheritance in Man.

We developed the survey according to the information needed from participants to evaluate usage, advantages, and limitations with a focus on privacy concerns. It included 3 demographic questions, 11 questions about frequency and details of group usage, 9 statements on positive/negative aspects and privacy concerns, and 3 statements on involvement of medical professionals. Opinions on attitudinal/opinion-based questions were elicited using a 5-point Likert scale or binary (yes/no) scale (Multimedia Appendix 1). The online survey was provided using SurveyMonkey [26]. Prior to participation, respondents were informed about the research project’s purpose and the voluntary and anonymous nature of their participation. Respondents were informed that withdrawal was possible at any given time and without consequences. No further incentive or reimbursement was given. Starting the questionnaire constituted informed consent to study participation.

By contacting 12 groups we expected to reach the target sample size of 100 respondents. This sample size was thought to provide a sufficient overview of usage data trends and the range of opinions about positive and negative aspects of Facebook usage for childhood rare disease support groups. Being a purely descriptive study, power calculations were not needed. Effectively, exceeding these expectations, a sample size of 238 respondents was reached, of whom 7 were excluded as the inclusion criterion question had not been answered.

The study and recruitment method have been reviewed by the Ethics Commission of Cologne University’s Faculty of Medicine (19-1027) and all research had been carried out within the scope of the approved study.

Survey answers were statistically analyzed by standard descriptive statistical methods using IBM SPSS statistics version 26. Metric data (age) were presented using mean and SD, ordinal data by the median and IQR, and binary and categorical variables using counts and percentages. Kendall τ was used to calculate rank correlations.

## Results

### Overview

In total, 231 respondents affirmed participation in a Facebook support group for a rare disorder; 7 did not respond to this question and were thus removed from the sample. Of the total respondents, 87.0% (n=201) were female, 12.6% (n=29) were male, and 0.4% reported another sex (n=1). Respondents’ mean age was 41.56 years (SD 9.375), with a median of 39 years (range 21-80 years) and an IQR of 10 years (Q1=35, median=39, Q3=45).

Of the 231 study respondents, 91.3% (n=211) were parents (183 mothers, 27 fathers, and 1 other sex), 5.6% (n=13) other relatives (eg, grandmothers, child), 1.7% (n=4) patients, 1 friend, 1 health professional, and 1 with no specified connection (0.4% each).

As much as 59.7% (138/231) reported a self-initiated search for the Facebook group, 24.2% (56/231) received recommendations from their health professionals, and 12.6% (29/231) recommendations from someone else affected by the disease. A total of 5/231 respondents created the group (2.2%), 1/231 respondent found the group via disease-related website (0.4%), and 1/231 via Facebook post (0.4%).

### Accessibility and Group Usage

On average, support group members visited Facebook at least once a day, visited and passively participated (read/liked posts) several times a week, and participated actively (commented/posted) once a month. Answers ranged from less than every 3 months to at least once a day for all questions (Table 2). Kendall τ showed a weak positive correlation between overall Facebook usage and support group usage (r=0.334, *P*<.0001).

**Figure 1:**
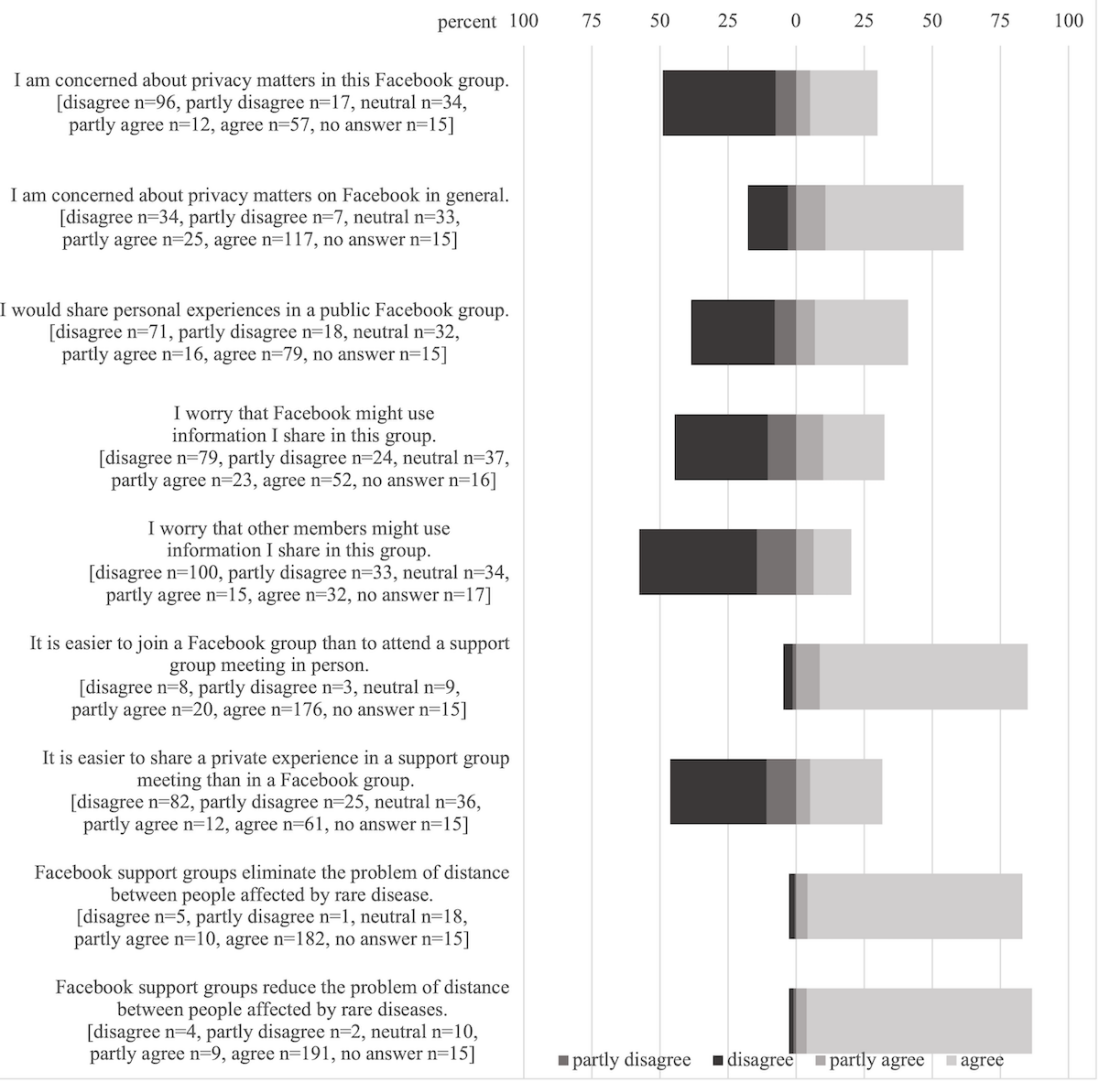
Group member's perceived benefits and concerns regarding Facebook support groups for pediatric rare diseases.

Most members used the Facebook group to find medical information about the disease, to read about personal experiences concerning the disease, to get advice on caring for someone with this disease, and to share their personal experiences concerning the disease. They agreed that Facebook reduces and eliminates the problem of distance between people affected by rare diseases (Figure 1).

**Table 2:** Facebook overall and Facebook support group usage frequencies (N=231).

Frequency	Overall Facebook usage, n (%)	Facebook support group usage, n (%)	Passive participation, n (%)	Active participation, n (%)
At least once a day	175 (75.8)	89 (38.5)	71 (30.7)	20 (8.7)
Several times per week	38 (16.5)	88 (38.1)	100 (43.3)	38 (16.5)
Once a week	7 (3.0)	31 (13.4)	34 (14.7)	41 (17.7)
Once a month	2 (0.9)	9 (3.9)	9 (3.9)	62 (26.8)
Once every 3 months	0 (0.0)	3 (1.3)	3 (1.3)	27 (11.7)
Less than once every 3 months	2 (0.9)	5 (2.2)	5 (2.2)	34 (14.7)
No answer	7 (3.0)	6 (2.6)	9 (3.9)	9 (3.9)
Total	231 (100.0)	231 (100.0)	231 (100.0)	231 (100.0)
Median	At least once a day	Several times per week	Several times per week	Once a month
IQR	0	Several times per week to at least once a day	Several times per week to at least once a day	Once every 3 months to several times per week

### Support Group Benefits

**Figure 2:**
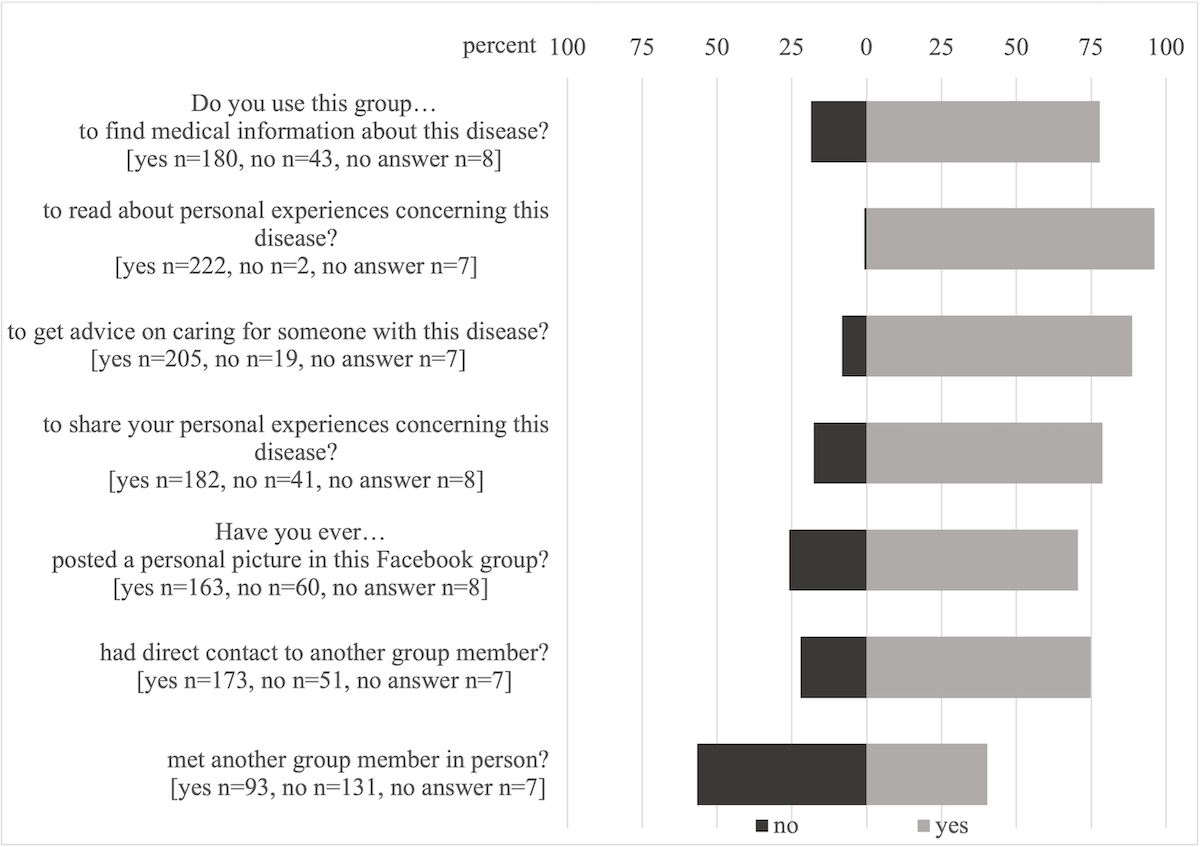
Facebook support group usage.

In our survey, we investigated 3 main categories of perceived benefits: informational support (finding medical information, getting advice on caring for someone with this disease), emotional support (reading or sharing personal experiences), and connecting with others (Figure 2).

### Privacy Perception and Concerns

Information shared in a public Facebook group is accessible to every Facebook user worldwide; however, a private group offers a more selected audience. Group members expressed more concern about privacy issues on Facebook in general than in their respective Facebook support groups, with concerns mostly related to Facebook itself and nongroup members. Twice as many respondents agreed to being concerned about privacy matters on Facebook in general than to being concerned about privacy matters in their Facebook group (Figure 1).

### Using Facebook for Communication With Health Professionals, Pharmaceutical Companies, and Study Recruitment

**Figure 3:**
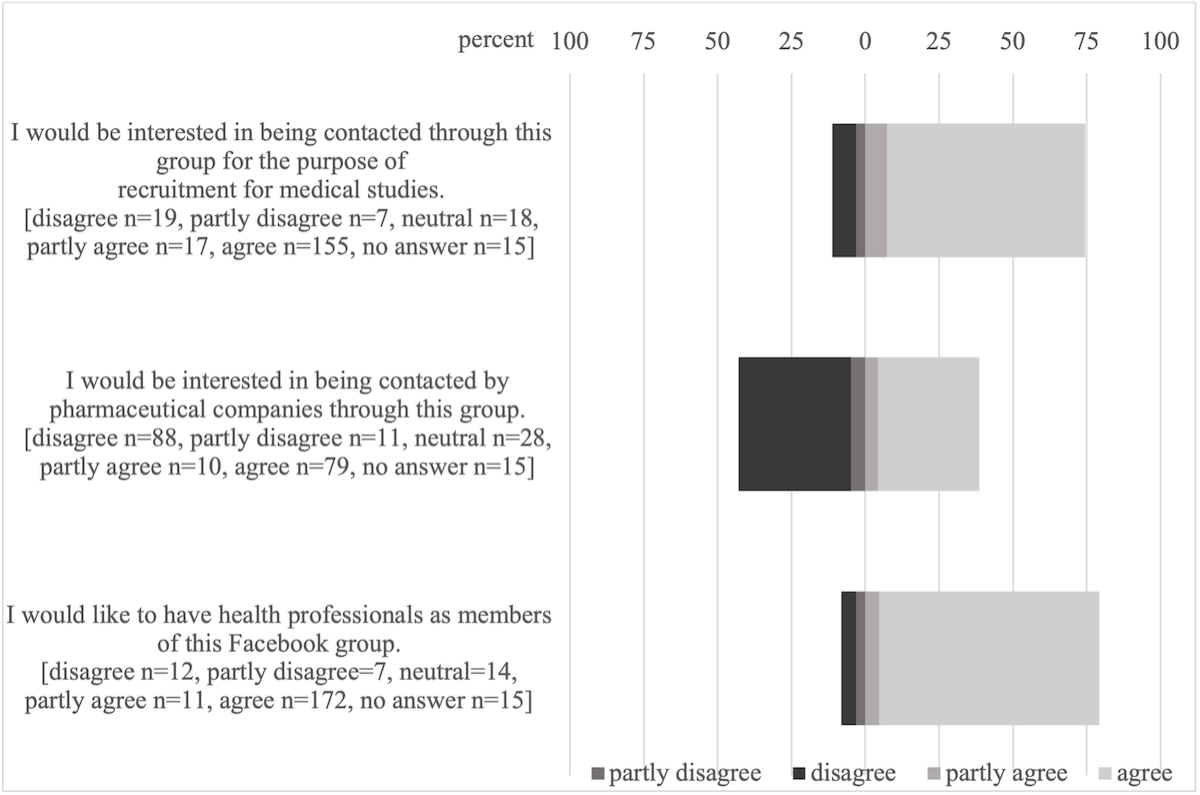
Group member’s perspective on involvement of health professionals, pharmaceutical companies and study recruitment in Facebook support groups for pediatric rare diseases.

Concerning group member’s perspectives on being contacted through their respective group, 67.1% (155/231) and 7.4% (17/231) fully and partly agreed, respectively, that they would be interested in being contacted through this group for the purpose of recruitment for medical studies and 34.2% (79/231) and 4.8% (11/231) fully and partly agreed, respectively, that they would be interested in being contacted by pharmaceutical companies. As much as 74.5% (172/231) and 4.8% (11/231) fully and partly agreed, respectively, that they would like to have health professionals as members of the respective Facebook group (Figure 3).

## Discussion

### Accessibility and Group Usage

Our study confirmed that Facebook enhances support group accessibility for parents of children with rare diseases. For most participants (196/231, 84.8%) it was easier to join a Facebook group than to attend a support group meeting in person. Reasons may include lack of in-person support group due to large geographic distances, limited time, and means of transportation. This increased accessibility is an important advantage specifically for the field of rare diseases with often large geographic distances between affected individuals.

Group members’ regular participation rates (Table 2) likely indicate high accessibility and practicality. Only 31.6% (73/231) of respondents agreed to finding it easier to share a private experience in a support group meeting than in a Facebook group, suggesting that Facebook support groups could possibly be considered an equally adequate setting for support groups. By providing options for both passive (read/like) and active (comment/post) participation, Facebook allows different degrees of involvement, which can facilitate group participation.

### Support Group Benefits

These 3 main categories of perceived benefit (informational support, emotional support, and connecting with others) are common benefit categories previously investigated in studies on pediatric rare diseases or online support [1,4-6,11,22-24].

#### Informational Support

As 77.9% (180/231) of respondents used their Facebook group to find medical information about the respective disease, our study strengthens previous studies’ findings that social media, including Facebook, is being used to search for medical information [5,6,11,22,23]. Increased knowledge can reduce stress related to parental incompetence and may result in better use of resources in the family [27]. Disease-specific Facebook support groups also facilitate the exchange of personal experiences in caring for someone affected; 88.7% (205/231) of respondents to our survey used their Facebook group to obtain advice on caretaking. This can ultimately improve parental management and care of rare conditions and have an empowering effect on parents who can become experts in their child’s care [11].

#### Emotional Support

Reading and sharing personal experiences is a starting point for emotional support in patient-to-patient communication. Seeing others’ similar experiences can install a sense of belonging [25], seeing same struggles can make one feel less alone, and seeing others’ positive developments can give hope. Almost all group members (222/231, 96.1%) used the Facebook group to read personal experiences and 78.8% (182/231) also shared personal experiences. About 70.6% (163/231) had already posted a personal picture in their group (Figures 1 and 2). Group participants’ frequent reading and sharing of personal experiences confirm that emotional support is a fundamental element of support groups, including support groups for rare diseases [4,6,22,24].

#### Connecting With Others

Facebook support groups and social media in general enable parents to connect with others [1,4,6,11,22,28]. This is especially important for those affected by rare diseases, because distance between affected individuals is a challenge in rare diseases which highly contributes to social isolation. Most respondents to our study agreed that Facebook reduces (200/231, 86.6%) and even eliminates (192/231, 83.1%) the problem of distance between people affected by rare diseases (Figure 2).

Facebook provides multiple communication functions including group and individual communication [7]. Usage of these functions is evident in our study: all respondents participated in group communication and 74.9% (173/231) reported direct contact to another group member via personal messaging services. Connections are also reported outside the virtual world: about 40.3% (9/231) had already met another group member in person (Figure 2). A study on online support groups for autism spectrum disorders indicated that a connection via Facebook could also be the starting point of organizing meetings for particular events [24].

These findings underline that parents and caregivers of children with rare diseases use Facebook support groups to connect and build relationships, and that Facebook is particularly useful for connecting with others affected by rare diseases by addressing the problem of distance between affected individuals.

### Privacy

Two-thirds of support groups for pediatric rare diseases on Facebook are private groups [9], reflecting on members’ need for privacy when sharing personal information and experiences online. In our survey, participants were divided about whether or not they would share personal experiences in a public group. About 41.1% (95/231) would share personal experiences in a public Facebook group, whereas 38.5% (89/231) would not, while 13.9% (32/231) remained neutral (Figure 1). These negative answers showed that privacy setting can be decisive for active support group participation. Privacy concerns generally appear to be mostly directed at Facebook itself and users who are not involved in the group: while only 29.9% (69/231) were concerned about privacy matters in their Facebook group, 61.5% (142/231) were concerned about privacy matters on Facebook in general and 32.5% (75/231) worried that Facebook might use information they shared in their group compared with 20.3% (47/231) who worried that other members might use this information (Figure 1). This suggests that their Facebook groups achieve a certain environment of perceived privacy.

As we have shown, most respondents of this study shared private information on Facebook, even though they had privacy concerns. This shows that the privacy paradox, which describes the discrepancy between expressed privacy concerns and actual information disclosure [18], is also applicable to pediatric rare disease support group members. More discussions on actual privacy, perceived privacy, and responsible decision making on online information disclosure with regard to protecting children’s privacy rights are needed. With an increasing number of Facebook support groups and increasing relevance for affected families, ultimately, guidelines on sharing children’s personal information online will be needed.

### Using Facebook for Communication With Health Professionals, Pharmaceutical Companies, and Study Recruitment

Giving recommendations to look for a Facebook group appears to be common practice, with 24.2% (56/231) of the participants having been referred to the Facebook group by a health professional (Figure 3). Having investigated the opportunities and limitations of Facebook support groups, our study can improve health professional’s knowledge on this type of support groups. When giving the recommendation to look for a support group on Facebook, health professionals can use this knowledge to inform individuals about the points that should be considered prior to joining a group, which include benefits that are to be expected, which prerequisites and limitations could possibly be encountered, and that sharing personal (health) information online requires careful consideration.

### Study Strengths and Limitations

Our study had a larger than expected sample size, with various diseases and support groups being represented. There were only few ethical implications because data were collected anonymously and respondents were given the option to omit questions if they did not feel comfortable answering. This may result in a low social desirability bias and central coherence bias. Nevertheless, representability and external validity can be questioned. Only groups addressing 12 pediatric rare diseases were invited, and our demographic analysis shows that males were under-represented. The study results might be influenced by a response/selection bias, because anyone completing the survey self-selected to do so, especially regarding the question on study recruitment. Future research should involve a more in-depth analysis of participant’s privacy concerns and behavior, including participant’s decision-making process on online information disclosure with regard to children’s privacy rights.

### Conclusion

We have shown that Facebook is a suitable tool for pediatric rare disease support groups, offering the distinct advantages of high accessibility and practicality. Group participants perceive a reduction and elimination of distance between affected individuals, a common challenge in rare disease, and Facebook support groups create an environment of perceived privacy allowing participants to share personal experiences and pictures.

We confirmed that participants of Facebook support groups for pediatric rare diseases benefit from informative support, emotional support, and the opportunity to connect with others. Our study has confirmed that most support group members use their Facebook group to find medical information, and further research is needed regarding how parents process and apply information found in online support groups to evaluate the risk of information inaccuracy and misinterpretation. Through our recruitment methods we provide an example of how Facebook support groups can be used for study recruitment and our survey showed that many group participants are in favor of study recruitment through their Facebook support groups.

We found that a group’s privacy setting can be a critical factor for active support group participation. Furthermore, we have shown the importance of discussing children’s privacy rights: sharing personal information and pictures on Facebook is very common among group participants. Group member’s privacy concerns appear to be mostly directed at Facebook itself and to users not involved in the group, which offers potential starting points for improving privacy in Facebook support groups. Our study showed that the privacy paradox is applicable to group members’ online information disclosure habits: parents share private information even though they are concerned about privacy matters on Facebook. Parents could benefit from guidance on responsible decision making about online information disclosure with regard to protecting children’s privacy rights. Ultimately, guidelines on sharing children’s personal information online could be a useful tool for finding the right balance between the risks of information disclosure and the benefits of participating in a support group on Facebook.

## Appendix

Multimedia Appendix 1 Questionnaire: Pediatric rare disease support groups on Facebook.
