# Supplementary material for: Facebook Support Groups for Pediatric Rare Diseases: Cross-Sectional Study to Investigate Opportunities, Limitations, and Privacy Concerns
Source: JMIR Pediatr Parent. 2022 Jan 6;5(1):e31411. doi: 10.2196/31411 (PMC8778561; doi:10.2196/31411)
Supplement: Multimedia Appendix 1 [file pediatrics_v5i1e31411_app1.docx]

| **Item No.** | **Question/Statement** | | **Answer** | | | | | | | | | | | | | | | | | | | | | |
| --- | --- | --- | --- | --- | --- | --- | --- | --- | --- | --- | --- | --- | --- | --- | --- | --- | --- | --- | --- | --- | --- | --- | --- | --- |
| 1 | Are you part of a Facebook support group for a rare disease? | | | | | | | | | | | yes | | | | | | | no | | | | | |
| 2 | Please select your age. | | 0-99 | | | | | | | | | | | | | | | | | | | | | |
| 3 | Please select your sex. | | female | | | | male | | | | | | | other | | | | | | | | | | |
| 4 | What is your connection to this rare disease? | | | patient | | parent | sibling | | other relative | | | | | friend | | | | health professional | | | | | | |
| 5 | How did you learn about this Facebook group? | self-initiated search for a group | | | recommendation by health professional | | | recommendation by someone else affected by this disease | | | | | | | | | other  (please specify) | | | | | | | |
|  | How often do you … a= more than once daily, b=once daily, c=several times per week,  d=once per week, e=once per month, f=less than once per month | | | | | | | | | | | | | | | | | | | | | | | |
| 6 | visit Facebook.com/ Facebook App, not including Facebook messenger? | | | | | | | | | a | b | | c | | | d | | | | e | | | f | |
| 7 | visit the Facebook group? | | | | | | | | | a | b | | c | | | d | | | | e | | | f | |
| 8 | read and/or like posts in this group? | | | | | | | | | a | b | | c | | | d | | | | e | | | f | |
| 9 | comment and/or post in this group? | | | | | | | | | a | b | | c | | | d | | | | e | | | f | |
|  | Do you use this Facebook group … | | | | | | | | | | | | | | | | | | | | | | | |
| 10 | to find medical information about this disease? | | | | | | | | | | | | | | yes | | | | | | no | | | |
| 11 | to read about personal experiences concerning this disease? | | | | | | | | | | | | | | yes | | | | | | no | | | |
| 12 | to get advice on caring for someone with this disease? | | | | | | | | | | | | | | yes | | | | | | no | | | |
| 13 | to share your personal experiences concerning this disease? | | | | | | | | | | | | | | yes | | | | | | no | | | |
|  | Have you ever… | | | | | | | | | | | | | | | | | | | | | | | |
| 14 | posted a personal picture in this Facebook group? | | | | | | | | | | | | | | yes | | | | | | no | | | |
| 15 | had direct contact to another group member via personal messaging services, e.g. Facebook messenger? | | | | | | | | | | | | | | yes | | | | | | no | | | |
| 16 | met another Facebook group member in person? | | | | | | | | | | | | | | yes | | | | | | no | | | |
|  | Please evaluate the following statements. 1=disagree, 2=partly disagree, 3=neutral, 4=partly agree, 5=agree (These questions and responses are purely hypothetical. This research is in no way connected to any other studies nor pharmaceutical companies. You will not be contacted after this survey.) | | | | | | | | | | | | | | | | | | | | | | | |
| 17 | I am concerned about privacy matters in this Facebook group. | | | | | | | | | | | | | | 1 | 2 | | | 3 | | | 4 | | 5 |
| 18 | I am concerned about privacy matters on Facebook in general. | | | | | | | | | | | | | | 1 | 2 | | | 3 | | | 4 | | 5 |
| 19 | I would share personal experiences in a public Facebook group. | | | | | | | | | | | | | | 1 | 2 | | | 3 | | | 4 | | 5 |
| 20 | I worry that Facebook might use information I share in this group. | | | | | | | | | | | | | | 1 | 2 | | | 3 | | | 4 | | 5 |
| 21 | I worry that other group members might use information I share in this group. | | | | | | | | | | | | | | 1 | 2 | | | 3 | | | 4 | | 5 |
| 22 | It is easier to join a Facebook group than to attend a support group meeting in person. | | | | | | | | | | | | | | 1 | 2 | | | 3 | | | 4 | | 5 |
| 23 | It is easier to share a private experience in a support group meeting than in a Facebook group. | | | | | | | | | | | | | | 1 | 2 | | | 3 | | | 4 | | 5 |
| 24 | Facebook support groups eliminate the problem of distance between people affected by rare diseases. | | | | | | | | | | | | | | 1 | 2 | | | 3 | | | 4 | | 5 |
| 25 | Facebook support groups reduce the problem of distance between people affected by rare diseases. | | | | | | | | | | | | | | 1 | 2 | | | 3 | | | 4 | | 5 |
| 26 | I would be interested in being contacted through this group for the purpose of recruitment for medical studies. | | | | | | | | | | | | | | 1 | 2 | | | 3 | | | 4 | | 5 |
| 27 | I would be interested in being contacted by pharmaceutical companies through this group. | | | | | | | | | | | | | | 1 | 2 | | | 3 | | | 4 | | 5 |
| 28 | I would like to have health professionals as members of this Facebook group. | | | | | | | | | | | | | | 1 | 2 | | | 3 | | | 4 | | 5 |
